# Supplementary material for: Reliability and responsiveness of a tissue hardness meter and algometer for measuring tissue hardness and pressure pain threshold in upper trapezius myofascial trigger points
Source: PeerJ. 2025 Jun 9;13:e19580. doi: 10.7717/peerj.19580 (PMC12161125; doi:10.7717/peerj.19580)
Supplement: Supplemental Information 3 [file peerj-13-19580-s003.doc]

GRRAS checklist for reporting of studies of reliability and agreement

Version based on Table I in: Kottner J, Audigé L, Brorson S, Donner A, Gajeweski BJ, Hróbjartsson A, Robersts C, Shoukri M, Streiner DL. Guidelines for reporting reliability and agreement studies (GRRAS) were proposed. J Clin Epidemiol. 2011;64(1):96-106

| **Section** | **Item #** | **Checklist item** | **Reported on page #** |
| --- | --- | --- | --- |
| Title/Abstract | 1 | Identify in title or abstract that interrater/intra rater  reliability or agreement was investigated. | 2  (Line 1; 18-20) |
| Introduction | 2 | Name and describe the diagnostic or measurement device of interest explicitly. | 4  (Line 50-67) |
|  | 3 | Specify the subject population of interest. | 5  (Line 94) |
|  | 4 | Specify the rater population of interest (if applicable). | NA |
|  | 5 | Describe what is already known about reliability and  agreement and provide a rationale for the study (if applicable). | 4-5  (Line 68-84) |
| Methods | 6 | Explain how the sample size was chosen. State the determined number of raters, subjects/objects, and replicate observations. | 6-7  (Line 109-128) |
|  | 7 | Describe the sampling method. | 9  (Line 179-180) |
|  | 8 | Describe the measurement/rating process (e.g. time interval between repeated measurements, availability  of clinical information, blinding). | 10  (Line 184-186) |
|  | 9 | State whether measurements/ratings were conducted independently. | 9  (Line 180-182) |
|  | 10 | Describe the statistical analysis. | 10-12  (Line 200-239) |
| Results | 11 | State the actual number of raters and subjects/objects  which were included and the number of replicate observations which were conducted. | 12: (Line 242-245)  14: (Line 277-280) |
|  | 12 | Describe the sample characteristics of raters and  subjects (e.g. training, experience). | 12: (Line 242-245)  14: (Line 277-280) |
|  | 13 | Report estimates of reliability and agreement including measures of statistical uncertainty. | 12-14  (Line 241-290) |
| Discussion | 14 | Discuss the practical relevance of results. | 16  (Line 325-331) |
| Auxiliary material | 15 | Provide detailed results if possible (e.g. online). | The journal’s website as a supplementary file. |
