# Supplementary material for: Reliability and responsiveness of a tissue hardness meter and algometer for measuring tissue hardness and pressure pain threshold in upper trapezius myofascial trigger points
Source: PeerJ. 2025 Jun 9;13:e19580. doi: 10.7717/peerj.19580 (PMC12161125; doi:10.7717/peerj.19580)
Supplement: Supplemental Information 4 [file peerj-13-19580-s004.docx]

# README: Variable Descriptions for R_TH_Reli.csv

| **Column Name** | **Description** | **Unit** |
| --- | --- | --- |
| ID | Participant ID number | - |
| Time | Time points of measurement (e.g., First, Second) | - |
| TH_WS_First | Tissue Hardness measured by Expert – First trial | % |
| TH_WS_Second | Tissue Hardness by Expert – Second trial | % |
| TH_WS_Third | Tissue Hardness by Expert – Third trial | % |
| TH_SO_First | Tissue Hardness by Beginner – First trial | % |
| TH_SO_Second | Tissue Hardness by Beginner – Second trial | % |
| TH_SO_Third | Tissue Hardness by Beginner – Third trial | % |
| PPT_WS_First | Pressure Pain Threshold by Expert – First trial | kg/cm² |
| PPT_WS_Second | Pressure Pain Threshold by Expert – Second trial | kg/cm² |
| PPT_WS_Third | Pressure Pain Threshold by Expert – Third trial | kg/cm² |
| PPT_SO_First | Pressure Pain Threshold by Beginner – First trial | kg/cm² |
| PPT_SO_Second | Pressure Pain Threshold by Beginner – Second trial | kg/cm² |
| PPT_SO_Third | Pressure Pain Threshold by Beginner – Third trial | kg/cm² |
| VAS | Pain intensity reported by participant using Visual Analog Scale | cm (0–10 scale) |
| Severity_VAS | Categorical pain severity rating (e.g., 1 = mild, 2 = moderate, 3=severe) | - |
