# Supplementary material for: Reliability and responsiveness of a tissue hardness meter and algometer for measuring tissue hardness and pressure pain threshold in upper trapezius myofascial trigger points
Source: PeerJ. 2025 Jun 9;13:e19580. doi: 10.7717/peerj.19580 (PMC12161125; doi:10.7717/peerj.19580)
Supplement: Supplemental Information 5 [file peerj-13-19580-s005.docx]

# README: Variable Descriptions for R_TH_GRC.csv

| **Column Name** | **Description** | **Unit** |
| --- | --- | --- |
| ID | Participant ID number | - |
| VAS_pre | Pain intensity before treatment (Visual Analog Scale) | cm (0–10) |
| Severity_VAS_pre | Categorical pain severity before treatment | Ordinal scale |
| VAS_post | Pain intensity after treatment (Visual Analog Scale) | cm (0–10) |
| Severity_VAS_post | Categorical pain severity after treatment | Ordinal scale |
| VAS_change | Change in VAS score (Post - Pre) | cm |
| NDI_pre | Neck Disability Index before treatment | Score (0–50) |
| Severity_NDI_pre | Categorical NDI severity before treatment | Ordinal scale |
| NDI_post | Neck Disability Index after treatment | Score (0–50) |
| Severity_NDI_post | Categorical NDI severity after treatment | Ordinal scale |
| NDI_change | Change in NDI score (Post - Pre) | Score |
| TH_pre | Tissue Hardness before treatment | % |
| TH_post | Tissue Hardness after treatment | % |
| TH_change | Change in Tissue Hardness (Post - Pre) | % |
| PPT_pre | Pressure Pain Threshold before treatment | Kg/cm^2^ |
| PPT_post | Pressure Pain Threshold after treatment | Kg/cm^2^ |
| PPT_change | Change in PPT (Post - Pre) | Kg/cm^2^ |
| GRC_PT | Physiotherapist-reported Global Rating of Change | Ordinal scale (-7 to +7) |
| GRC_pt | Patient-reported Global Rating of Change | Ordinal scale (-7 to +7) |
